# STC1‐Based Activation of NF‐κB Signaling Pathway Induces Epthithelial–Mesenchymal Transition Thus Promotes Progression and Temozolomide Resistance of Glioblastoma

**DOI:** 10.1096/fj.202500095R

**Published:** 2025-09-29

**Authors:** Jia Wang, Beichen Zhang, Haoyu Zhou, Bin Liu, Xiaobin Bai, Wei Wu, Ruichun Li, Wanfu Xie

**Affiliations:** ^1^ Department of Neurosurgery The First Affiliated Hospital of Xi'an Jiaotong University Xi'an Shaanxi China; ^2^ Center of Brain Science The First Affiliated Hospital of Xi'an Jiaotong University Xi'an Shaanxi China; ^3^ Department of Neurosurgery Qinghai Provincial Peoples Hospital Xining Qinghai China

**Keywords:** EMT, glioblastoma, metastasis, NF‐κB, STC1

## Abstract

Acquired resistance to chemotherapy, especially to temozolomide (TMZ), is a major challenge correlated with the treatment failure of glioblastoma (GBM). Stanniocalcin‐1 (STC1) is a glycoprotein hormone involved in multiple biological processes in cancer cells. However, the function and underlying mechanism of STC1 in GBM still remain unclear. To this end, exploring the potential functional role and mechanism of STC1 inducing TMZ resistance becomes an urgent need for individual individualized strategies for GBM. The GSE151680 dataset was obtained from the GEO database; thus, bioinformatic analysis was performed by using R software (version 4.2.0) to screen the differentially expressed genes correlated to TMZ resistance in GBM. Cox regression and nonnegative matrix factorization (NMF) analysis were conducted to establish a prognostic model. Additionally, immunohistochemistry (IHC) staining, qRT‐PCR, and western blot were used to investigate the expression of STC1 in GBM tissues and non‐tumor controls. Mechanically, loss‐of‐function and gain‐of‐function assays were performed to validate the biological functions of STC1 on the malignant biological characters and TMZ resistance of GBM cells. Besides, the enrichment analysis was performed to investigate the downstream pathway of STC1. In this study, STC1 was selected as the gene candidate correlated to TMZ resistance according to the results of Cox regression and NMF analysis. Additionally, increased expression of STC1 could be observed in GBM and was significantly correlated to poor prognosis in GBM. Besides, multiple malignant characters including proliferation, migration, invasion, tumorigenesis, and TMZ resistance of GBM could be markedly reduced by exogenous downregulation of STC1; contrarily, overexpression of STC1 promoted the malignant behaviors and drug resistance of GBM cells. Moreover, GO, KEGG, and GSEA analysis revealed that STC1 induced epithelial‐mesenchymal transition (EMT) via activation of NF‐κB signaling. Furthermore, the treatment of TNF‐α (an activator of the NF‐κB pathway) partially reversed the inhibitory effect of sh‐STC1 on the proliferation and metastasis in GBM cells. In conclusion, STC1 induced EMT thus enhances the malignancies and drug resistance of GBM cells by activating the NF‐κB pathway, providing new evidence for clinical drug development in GBM.

## Graphical abstract

Glioblastoma (GBM), a neuroepithelial‐derived lethal malignancy, remains incurable due to its intrinsic heterogeneity, necessitating molecular biomarkers for precision therapeutics. STC1 promotes GBM progression and temozolomide (TMZ) resistance by activating the NF‐κB signaling pathway, which drives epithelial–mesenchymal transition (EMT) and enhances tumor aggressiveness. Genetic or pharmacological inhibition of STC1 suppresses NF‐κB‐mediated EMT, reduces tumor invasion, and restores TMZ sensitivity in GBM models. Targeting the STC1/NF‐κB/EMT axis represents a promising therapeutic strategy for overcoming chemoresistance in GBM.

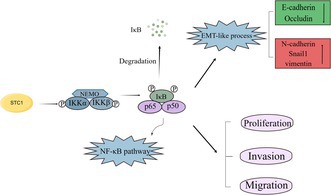

## 1 Introduction

Glioblastoma is the most aggressive and lethal primary brain tumor, characterized by rapid proliferation, extensive invasion, and resistance to conventional therapies, especially profound intratumoral and intertumoral heterogeneity [1]. Despite multimodal treatment strategies, including surgical resection, radiotherapy, and chemotherapy, the median survival of GBM patients remains dismal, typically less than 15 months [2]. The inherent heterogeneity of glioblastoma, manifested at genetic, epigenetic, cellular, and molecular levels, contributes to its ability to evade immune surveillance, develop therapeutic resistance, and drive tumor progression [3, 4]. This multifaceted complexity underscores the urgent need to identify novel molecular targets and develop innovative therapeutic strategies to overcome treatment resistance and improve patient outcomes.

Temozolomide (TMZ), a lipophilic alkylating prodrug, is activated to 3‐methyl‐(triazen‐1‐yl) imidazole‐4‐carboxamide (MTIC), which generates methyl diazonium cations that methylate DNA at O6‐guanine (O6‐MeG), inducing cytotoxic double‐strand breaks [5, 6, 7, 8]. However, TMZ efficacy is limited by DNA repair mechanisms, particularly O6‐methylguanine‐DNA methyltransferase (MGMT)‐mediated direct reversal of O6‐MeG lesions [9, 10]. Mismatch and base excision repair pathways further contribute to resistance [11, 12]. Current MGMT‐focused strategies often fail due to resistance heterogeneity [13], highlighting the need for novel multimodal biomarkers to overcome TMZ resistance in GBM.

Stanniocalcin‐1 (STC1) is a multifunctional glycoprotein involved in calcium homeostasis and tumor progression [14, 15, 16]. Previous studies have confirmed that STC1 promotes metastasis through EMT activation in ovarian cancer [17] and enhances cell survival via NF‐κB signaling in cervical cancer [18]. Substantial studies demonstrated that STC1 overexpression drives tumor aggressiveness through NOTCH1/SOX2 and TGF‐β pathways in GBM [19, 20], while also contributing to angiogenesis [21] and temozolomide (TMZ) resistance via MGMT regulation [22].

The NF‐κB pathway plays a central role in GBM pathogenesis, promoting TMZ resistance [23] and EMT‐mediated invasion [24]. While our previous work established NF‐κB‐EMT crosstalk in GBM progression [23, 25, 26], the upstream regulators linking this axis to chemoresistance remain unclear.

Here, we posit STC1 as a multimodal rheostat of GBM aggressiveness and chemoresistance. Bioinformatics analyses reveal STC1 overexpression correlates with advanced pathology and dismal prognosis. Functional studies demonstrate that STC1 genetic manipulation affects proliferation, invasion, and tumorigenesis, while its overexpression confers TMZ resistance in vitro and in vivo. Mechanistically, STC1 activates NF‐κB signaling to drive EMT, thereby synchronizing metastatic potential with therapy tolerance. Crucially, NF‐κB inhibition reverses STC1‐mediated EMT and resensitizes GBM cells to TMZ.

Our work delineates STC1 as a master regulator coupling NF‐κB‐driven EMT to TMZ resistance, providing a therapeutic nexus to dismantle GBM resilience. By transcending singular resistance mechanisms, this STC1‐NF‐κB‐EMT axis offers a multifaceted target to improve therapeutic outcomes in this recalcitrant malignancy.

## 2 Materials and Methods

### 2.1 Tissue Samples

All the tumor tissues and adjacent normal tissues were from GBM patients treated in The First Affiliated Hospital of Xi'an Jiaotong University, Xi'an, China. All glioblastoma tissue samples were obtained with the approval of the Scientific Ethics Committee at the First Affiliated Hospital of Xi'an Jiaotong University (approve no. 2021–695). Furthermore, patients provided written consent to participate in the study and to allow their samples to be used for research purposes.

### 2.2 Bioinformatics Analysis

The GSE151680 dataset was downloaded from the GEO database (https://www.ncbi.nlm.nih.gov/geo). Then, the data were treated and analyzed using R software (version 4.2.0). The expression difference of individual genes was identified by Log2 (Fold change) and adjusted *p* value, in which Log_2_FC > 1 with an adjusted *p* value < 0.05 was identified as a differentially expressed gene (DEG).

### 2.3 Construction of the Prognostic Model and Predictive Performance Evaluation

The prognostic model was conducted through Cox regression analysis using the survival R package, in which the top 20 prognosis‐related risk genes were presented and further evaluated by the Brier score value and the SHapley Additive exPlanations value (SHAP). These GBM samples were divided into two groups according to the overall survival using the nonnegative matrix factorization (NMF) algorithm, in which the filtered prognostic risk genes were used to construct the model. Then, the DEGs between the two groups were calculated by the limma package.

### 2.4 Immunohistochemistry (IHC) Staining

The tumor tissues of GBM were fixed using formalin solution and prepared into sections using paraffin‐embedded tissues. The tissues were deparaffinized, rehydrated, and incubated with mouse anti‐STC1 antibody (1:500, ab239518, USA) at 4°C overnight. Then, the samples were incubated with secondary antibodies at 37°C for 3 h, and color development was performed using DAB solution. Subsequently, the tissues were restrained using hematoxylin, and the results were observed by microscope.

### 2.5 Cell Culture

The GBM cell lines (U87, LN229, and A172) and normal human astrocyte (NHA) cell line from the American Type Culture Collection (ATCC, USA) were cultured in Dulbecco's Modified Eagle's media (DMEM, Gibco, China) containing 10% fetal bovine serum (FBS) and 1% penicillin/streptomycin at 37°C with 5% CO_2_.

### 2.6 Cell Transfection and Treatment

The lentivirus overexpressing/knockdown STC1 was designed and constructed by GenePharma (Shanghai, China). GBM cells in the logarithmic growth phase were trypsinized and made into single‐cell suspensions. Added the transfection reagents Lipofectamine 2000 (Beyotime, Shanghai, China) and plasmids into the medium after the cells had grown to 80% confluence, followed by the transfection efficiency was detected. Then, cells were treated with TMZ.

### 2.7 qRT‐PCR

Total RNA was extracted from GBM cell lines and tissues, and subsequently, the cDNA was obtained using the PrimeScript RT Master Mix (Takara, Kusatsu, Japan). Then, cDNA was used as a template for PCR amplification. The 2^−ΔΔCt^ method was applied to evaluate the relative mRNA expression. The primer sequences were listed as below:

STC1, #1 forward 5′‐CTGAAGTGGTTCGTTGCCTC‐3′ and reverse 5′‐CTGAGTGTCAAATTTAGCAGCG‐3′; #2 forward 5′‐AGGTGCAGGAAGAGTGCTACA‐3′ and reverse 5′‐GACGACCTCAGTGATGGCTT‐3′; GAPDH, forward 5′‐ACCCAGAAGACTGTGGATGG‐3′ and reverse 5′‐TTCAGC TCAGGGATGACCTT‐3′; N‐cadherin, forward 5′‐TCAGGCGTCTGTAGAGGCTT‐3′ and reverse 5′‐ATGCACATCCTTCGATAAGACTG‐3′; Snail1, forward 5′‐TCGGAAGCCTAACTACAGCGA‐3′ and reverse 5′‐AGATGAGCATTGGCAGCGAG‐3′; Vimentin, forward 5′‐AGTCCACTGAGTACCGGAGAC‐3′ and reverse 5′‐CATTTCACGCATCTGGCGTTC‐3′; E‐cadherin, forward 5′‐CGAGAGCTACACGTTCACGG‐3′ and reverse 5′‐GGGTGTCGAGGGAAAAATAGG‐3′; Occludin, forward 5′‐CGAGAGCTACACGTTCACGG‐3′ and reverse 5′‐GGGTGTCGAGGGAAAAATAGG‐3′.

### 2.8 Western Blot

The RIPA buffer (Qiagen, NRW, Germany) was used to lyse samples, and the BCA protein assay kit (Thermo Fisher Scientific, MA, USA) was used to analyze the protein concentration. Each protein sample was subjected to 10% SDS‐PAGE. The protein was transferred to PVDF membranes (Millipore, MA, USA), and then the membranes were incubated with 5% skimmed milk for 1 h at room temperature. Subsequently, the membranes were incubated overnight at 4°C with primary antibodies. Following this, the PVDF membrane was washed three times with TBST (Thermo Fisher Scientific, MA, USA) and incubated with HRP‐conjugated secondary antibody (1:5000) for 1 h at 37°C. The Amersham ECL Western Blot System (Cytiva) was applied to visualize the protein expression levels of each sample, using GAPDH as the loading control. The following antibodies were used: primary antibodies against STC1 (1:2000, ab239518, USA), E‐cadherin (1:1000, ab314063, USA), N‐cadherin (1:1000, ab76011, USA), Occludin (1:2000, ab216327, USA), Snail1 (1:1000, ab31787, USA), Vimentin (1:1000, ab92547, USA), IKKα (1:1000, ab32041, USA), P‐IKKα (1:1000, ab17943, USA), IκBα (1:1000, ab32518, USA), p65 (1:1000, ab32536, USA), and GAPDH (1:5000, ab8245, USA). Additionally, Anti‐Rabbit IgG and Anti‐Mouse‐IgG were purchased from Cell Signaling Technology (cat. no. #7074 and #7076, respectively).

### 2.9 EdU Staining

The EdU assay kit (Servicebio, Wuhan, China) was utilized to conduct EdU staining. Cells were cultured in the medium containing EdU solution. Two hours later, cells were fixed with 4% formaldehyde for 15 min. Cells were treated with 0.5% Triton X‐100 in PBS solution and stained with iF555 and Hoechst 33342. The stained cells were observed using a fluorescence microscope.

### 2.10 CCK‐8 Assay

The GBM cells were inoculated into 96‐well plates, and then the appropriate amount of CCK‐8 reagent (Dojindo Chemical Laboratory, Kumamoto, Japan) was added to each well. The plates were incubated in an incubator to fully react the cells with the CCK‐8 reagent. The OD values were measured at 450 nm.

### 2.11 Wound Healing Assay

The GBM cells in the logarithmic growth phase were digested into single‐cell suspensions and inoculated into a 6‐well plate. Then, we incubated the cells to a monolayer‐fused state and used a sterile lance tip to create a linear scratch on the cell monolayer. PBS buffer (Thermo Fisher Scientific, MA, USA) was used to wash the cells to remove cell debris and residue shed during the scratching process. The treated plate was placed in an incubator and continued to culture. The changes in the scratched area were observed using an inverted microscope (Olympus Corporation, Tokyo, Japan), and the migration of the cells was recorded.

### 2.12 Transwell Assay

GBM cells were cultured to the logarithmic growth phase, digested, centrifuged, and resuspended in a serum‐free medium. The appropriate amount of cell suspension was added to the upper chamber of the transwell, and serum‐containing medium was added to the lower chamber. Twenty‐four hours later, the transwell was removed, and the cells were fixed and stained using crystal violet (Merck, MA, USA). The GBM cells that migrated to the lower chamber were observed and counted under the microscope (Thermo Fisher Scientific, MA, USA).

### 2.13 Colony Formation Assay

The monolayer cells in the logarithmic growth phase were obtained to count the cells. After that, the GBM cells were inoculated into 6‐well plates at 200 cells per well and incubated in an incubator for 2 weeks. At the end of the specified incubation period, the formation of colonies was observed under a microscope, and the number of colonies formed in each well was recorded.

### 2.14 Intracranial Xenograft Tumor Model

Animals in this study was approved by the Ethics Committee of the School of Medicine, Xi'an Jiaotong University (approval no. 2021‐695). All the nude mice in this study were chosen randomly. Six‐week‐old female nude mice were used for the intracranial xenograft tumor model. The U87 or LN229 cells transduced with or without lentivirus (1 × 10^5^ cells in 2 μL PBS) were slowly injected into the brains of the nude mice as previously described (25921812). Five mice were used for each group. The mice were sacrificed and perfused with ice‐cold PBS and 4% (wt/vol) paraformaldehyde (PFA) when the following symptoms were observed: unsteady gait, arched back, more than 10% weight loss, or leg paralysis. The mice brains were subsequently dissected and fixed in 4% PFA for 24 h, transferred to 10% formalin, and sectioned. Bioluminescence imaging was performed to assess the growth of intracranial tumor.

### 2.15 Enrichment Analysis

The expression profiles of GBM samples in the TCGA database were divided into two groups based on the expression of STC1. The DEGs between the two groups were identified using “limma” package, and then were conducted the enrichment analysis was conducted. GO and KEGG analyses were used to investigate STC1‐related candidate pathways, while GSEA analysis was used as validation.

### 2.16 Statistical Analysis

To ensure the reliability of the results, all experiments were replicated across a minimum of three distinct groups. When comparing just two groups, we relied on the *t*‐test. However, for assessing disparities among multiple groups, we adopted one‐way ANOVA followed by Tukey's post hoc test. Statistical significance was defined as *p* < 0.05.

## 3 Results

### 3.1 Identification of EDGs Associated With TMZ Resistance in GBM Cell Lines

**FIGURE 1:**
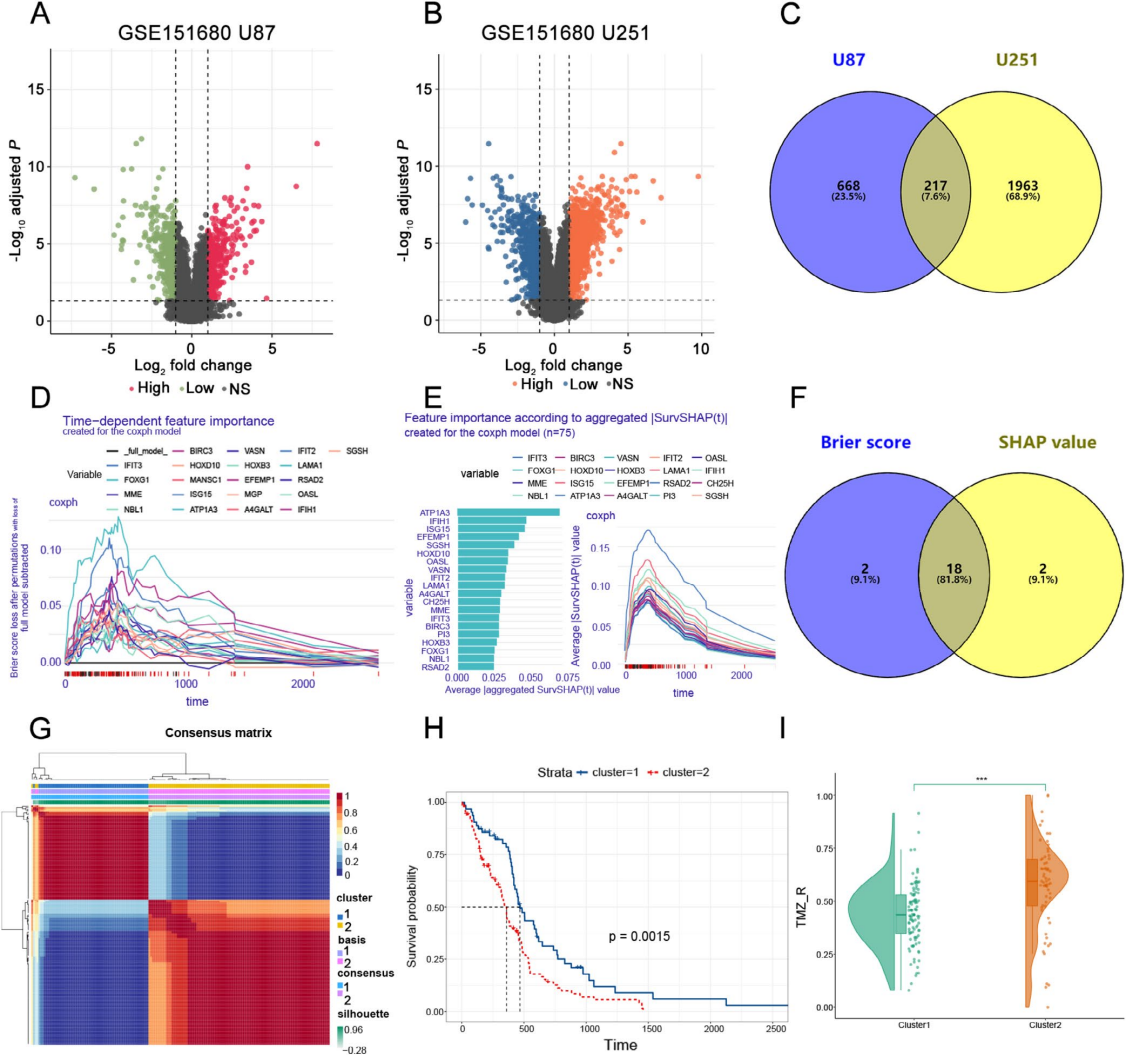
The differential expression genes (DEGs) in GSE151680 datasets. DEGs in TMZ‐sensitive and TMZ‐resistant U87 cell line (A) and U251 cell line (B). (C) Overlapping DEGs in the U87 and U251 cell lines. The Brier value (D) and SHAP value (E) for the top 20 genes in the Cox regression analysis. (F) Overlapping DEGs were screened based on Brier value and SHAP value. (G) NMF clustering based on 18 genes. (H) The Kaplan–Meier analysis of overall survival in two groups of GBM patients (*p* = 0.0015, with log‐rank test). (I) TMZ resistance score in two groups of GBM patients.

The data from the GEO database were analyzed to screen DEGs which were correlated to TMZ resistance in U87 and U251 naïve or TMZ‐resistant cell lines using the limma software package and visualized using a volcano graph (Figure 1A,B). Then, we obtained 217 DEGs by taking the intersection of U87 and U251 cell lines (Figure 1C). To further narrow the scope of the candidate genes, Cox regression analysis was conducted depending on different variables (Figure 1D,E). Based on the Brier value and SHAP value, we finally acquired 18 DEGs (Figure 1F). Then, the GBM patients' samples were divided into two groups based on the 18 genes mentioned above (Figure 1G). Kaplan–Meier curves analysis implied that two groups of patients had different prognoses (Figure 1H). Furthermore, this study also found that two groups of patients had different TMZ resistance scores calculated by ssGSEA, suggesting that GBM was clinically heterogeneous (Figure 1I, *p* = 0.0015).

### 3.2 Elevated STC1 Expression Indicated the Worst Outcomes in GBM

**FIGURE 2:**
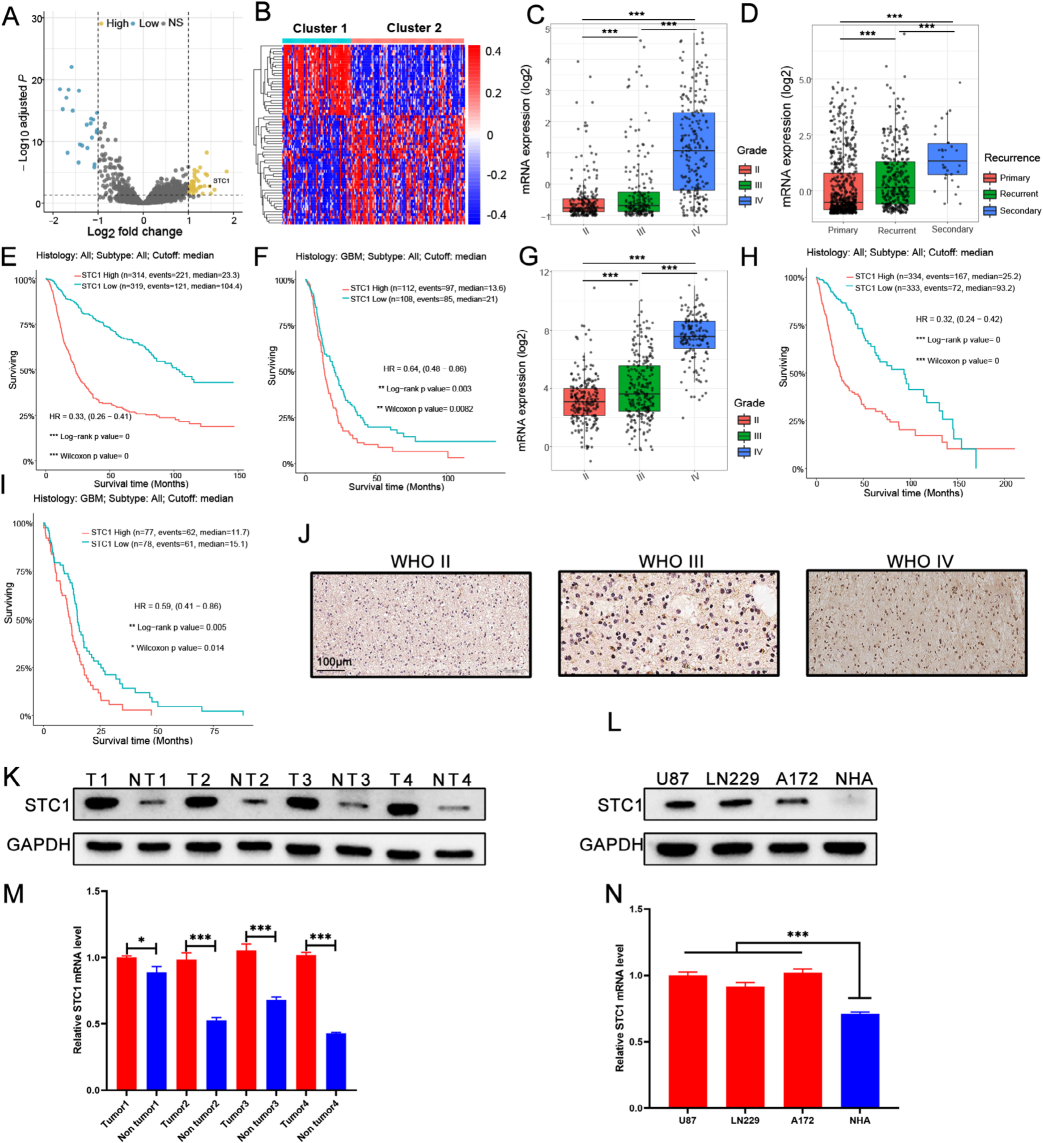
Elevated STC1 expression indicated the worse outcomes in GBM. The DEGs in GBM patients with different outcomes were displayed by a volcano graph (A) and a heatmap (B). The expression of STC1 in glioma according to WHO classification (C) or tumor recurrence (D) using the CGGA database (***all *p* < 0.001, HSD test). (E) The Kaplan–Meier analysis of overall survival in patients with glioma based on STC1 expression, using the CGGA database (*p* < 0.05, with log‐rank test). (F) The Kaplan–Meier analysis of overall survival in patients with GBM based on STC1 expression, using the CGGA database (*p* = 0.003, with log‐rank test). (G) The expression of STC1 in glioma according to WHO classification using the TCGA database (***all *p* < 0.001, HSD test). (H) The Kaplan–Meier analysis of overall survival in patients with glioma based on STC1 expression, using the TCGA database (*p* < 0.05, with log‐rank test). (I) The Kaplan–Meier analysis of overall survival in patients with GBM based on STC1 expression, using the TCGA database (*p* = 0.006, with log‐rank test). (J) Immunohistochemistry staining was performed to assess the expression of STC1 in different grades of glioma patients. (K) The protein expression of STC1 in tumor tissues and non‐tumor tissues. (L) The protein expression of STC1 in GBM and human astrocyte cell lines. (M) The mRNA expression of STC1 in tumor tissues and non‐tumor tissues (*p < 0.05, ***p < 0.001, with Student's *t* test, *n* = 3). (N) The mRNA expression of STC1 in GBM and human astrocyte cell lines (***p < 0.001, with Student's *t* test, *n* = 3).

The limma package was used to analyze the DEGs between the two groups of GBM patients with different prognoses mentioned above. The result was exhibited using a volcano graph (Figure 2A) and a heatmap (Figure 2B), as well as we noted that STC1 was notably upregulated in GBM patients with poor prognosis. Afterwards, the expression of STC1 was determined in different grades and different subtypes of GBM using the CGGA database. STC1 was upregulated in GBM patients compared with the low‐grade glioma patients (Figure 2C). Besides, the expression of STC1 was also closely related to the tumor recurrence, and patients with high STC1 had a higher recurrence rate (Figure 2D). Then, Kaplan–Meier survival suggested that glioma patients with higher expression of STC1 exhibited worse outcomes (Figure 2E,F, all *p* < 0.05). We analyzed the data from the TCGA database using the same methodology and also found that the expression of STC1 was higher in GBM compared with the other gliomas, as well as glioma patients with higher expression of STC1 had poor prognoses (Figure 2G–I, all *p* < 0.05). Moreover, we also assessed the expression of STC1 in GBM tissues collected from our hospital. The results indicated that the expression of STC1 was increased in glioma tissues along with the pathological characteristics (Figure 2J). Western blot and RT‐qPCR also confirmed that the protein and mRNA expression of STC1 was overexpressed in GBM tissues (Figure 2K,M, all *p* < 0.05). Indeed, the mRNA and protein expression of STC1 was also increased in GBM cell lines (Figure 2L,N, *p* < 0.001). Altogether, elevated STC1 implied the worse outcome of GBM patients.

### 3.3 Knockdown of STC1 Inhibited the Malignant Behaviors in GBM Cells

**FIGURE 3:**
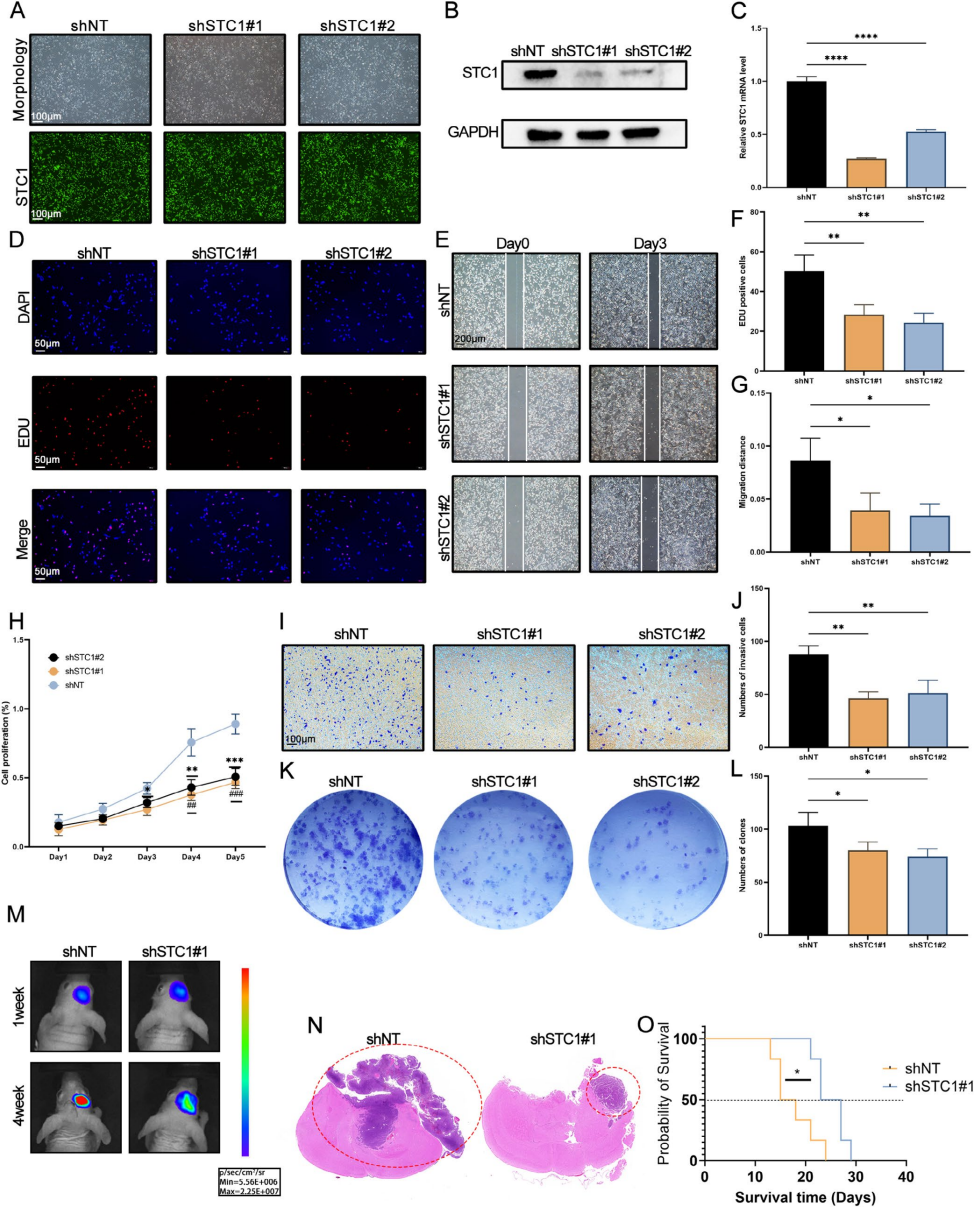
The knockdown of STC1 inhibited the malignant behaviors of GBM cells. GBM cells were transfected with lentiviral vectors expressing shRNA targeting STC1#1 (shSTC1#1), STC1#2 (shSTC1#2), or a scrambled control (shNT). (A) Representative fluoresce images in GBM cells (Upper panel: Morphology under microscope, Lower panel: Fluoresce images). Western blot (B) and qRT‐PCR (C) were used to determine the expression of STC1 (***p < 0.001, with Student's *t* test, *n* = 3). (D, F) EdU assay was used to assess the proliferation of GBM cells (**p < 0.01, with Student's *t* test, *n* = 3). (E, G) Wound healing assay was used to measure the migration of GBM cells (*p < 0.05, with Student's *t* test, *n* = 3). (H) CCK‐8 assay was used to detect the proliferation of GBM cells (*p < 0.05, **p < 0.01, ***p < 0.001, shNT vs. shSTC1#1, Student's *t* test, *n* = 3; ##*p* < 0.01, ###*p* < 0.001, shNT vs. shSTC1#2, Student's *t* test, *n* = 3). (I, J) Transwell invasion assay was utilized to detect the invasion of GBM cells (**p < 0.01, with Student's *t* test, *n* = 3). (K, L) Colony formation test was performed to assess the proliferation of GBM cells (*p < 0.05, with Student's *t* test, *n* = 3). (M) The tumor volume and fluorescence intensity values of mouse brains after the intracranial transplantation. (N) Representative H&E‐stained images for in vivo intracranial xenografted mice. (O) Kaplan–Meier survival analysis of the xenograft mice in the control group and STC1 knock‐down group (*n* = 5 in each group; *p < 0.05, with log‐rank test).

**FIGURE 4:**
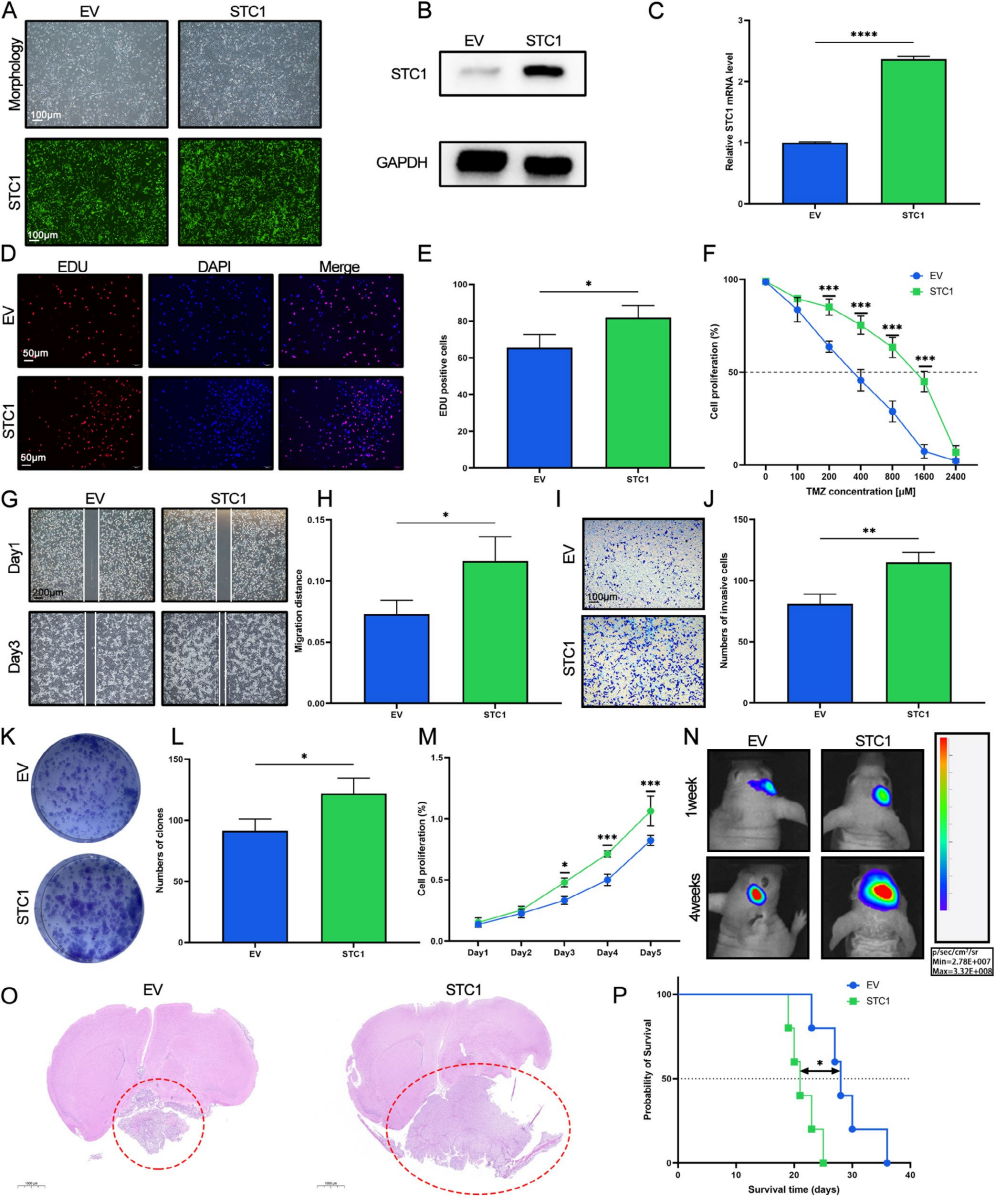
The overexpression of STC1 promoted the malignant biological behaviors and increased TMZ resistance in GBM cells. GBM cells were transfected with lentiviruses targeting STC1 and then treated with TMZ. (A) Representative fluoresces images in GBM cells (Upper panel: Morphology under microscope, Lower panel: Fluoresces images). Western blot (B) and qRT‐PCR (C) were used to detect the expression of STC1 (****p < 0.0001, with Student's *t* test, *n* = 3). (D, E) EdU assay was used to assess the proliferation of GBM cells (*p < 0.05, with Student's *t* test, *n* = 3). (F) CCK‐8 assay was used to detect the proliferation of GBM cells after treatment with TMZ (***p < 0.001, with Student's *t* test, *n* = 3). (G, H) Wound healing assay was used to measure the migration of GBM cells (*p < 0.05, with Student's *t* test, *n* = 3). (I, J) Transwell invasion assay was utilized to detect the invasion of GBM cells (**p < 0.01, with Student's *t* test, *n* = 3). (K, L) Colony formation test was performed to assess the proliferation of GBM cells (*p < 0.05, with Student's *t* test, *n* = 3). (M) CCK‐8 assay was used to detect the proliferation of GBM cells (*p < 0.05, ***p < 0.001, with Student's *t* test, *n* = 3). (N) The tumor volume and fluorescence intensity values of mouse brains after the intracranial transplantation. (O) Representative H&E‐stained images of mouse brain sections after the intracranial transplantation. Scale bars: 1 mm. (P) Kaplan–Meier survival curves of the xenograft mice in the control group and STC1 overexpression group (*p* < 0.05, with log‐rank test).

To further validate the effect of STC1 on GBM cell function, we constructed the lentiviral vectors targeting STC1 and transfected them into U87 cells. The efficiency of transfection was assessed through fluorescence (Figure 3A). The results of qRT‐PCR and western blot further suggested that the expression of STAC was decreased in STC1 knock‐down GBM cells (Figure 3B,C, *p* < 0.0001). Subsequently, we conducted the EdU and CCK‐8 assays to measure the proliferation of GBM cells. The knockdown of STC1 inhibited the proliferation of GBM cells (Figure 3D,F,H, all *p* < 0.05). Besides, wound healing and transwell invasion assays indicated that the deletion of STC1 also suppressed the migration and invasion of GBM cells (Figure 3E,G, *p* < 0.05). The transwell assay was performed to measure the invasion of GBM cells. The knockdown of STC1 inhibited the invasion of GBM cells (Figure 3I,J, *p* < 0.01). The results of the colony formation assay also indicated that the inhibition of STC1 reduced the proliferation of GBM cells (Figure 3K,L, *p* < 0.05). We chose the shSTC1#1 sequence to conduct the next experiment in vivo because of the knock‐down efficiency and better results. An intracranial xenografted mouse model was established to identify the function of STC1 in tumorigenesis in vivo. We found that downregulation of STC1 expression reduced the tumorigenesis of the glioblastoma cells (Figure 4M–O). Altogether, these results demonstrated that the knockdown of STC1 inhibited the malignant behaviors of GBM cells.

### 3.4 Overexpression of STC1 Promoted the Malignant Behaviors in GBM Cells

To further investigate the function of STC1 in LN229 GBM cells, gain‐of‐function experiments were conducted. As shown in Figure 4A, the efficiency of transfection was detected by fluorescence. Western blot and qRT‐PCR also revealed that GBM cells stably overexpressing STC1 were established successfully (Figure 4B,C, *p* < 0.0001). The EdU and CCK‐8 assays indicated that the proliferation of GBM cells was promoted in GBM cells overexpressing STC1 compared to normal GBM cells (Figure 4D,E,M, all *p* < 0.05). Furthermore, transwell and wound healing assays were utilized to detect the migration and invasion of GBM cells. The results revealed that STC1 advanced the migration and invasion of GBM cells (Figure 4G–J, all *p* < 0.05). Colony formation tests further verified that increased STC1 promoted the proliferation of GBM cells (Figure 4K,L, *p* < 0.05). Besides, we also found that overexpression of STC1 significantly promoted the proliferation of GBM cells after being treated with TMZ, indicating that STC1 overexpression increased the TMZ resistance in GBM cells (Figure 4F, *p* < 0.001). To investigate the function of STC1 in vivo, the GBM intracranial xenograft models were established. As expected, the results indicated that overexpression of STC1 increased the volume of the tumor and reduced the survival time of mice when compared with the control group (Figure 4N–P, *p* < 0.05). All in all, these results suggested that STC1 significantly contributes to the malignant biological behaviors of tumor cells and resistance to TMZ.

### 3.5 Knockdown of STC1 Inhibited EMT in GBM Cells

**FIGURE 5:**
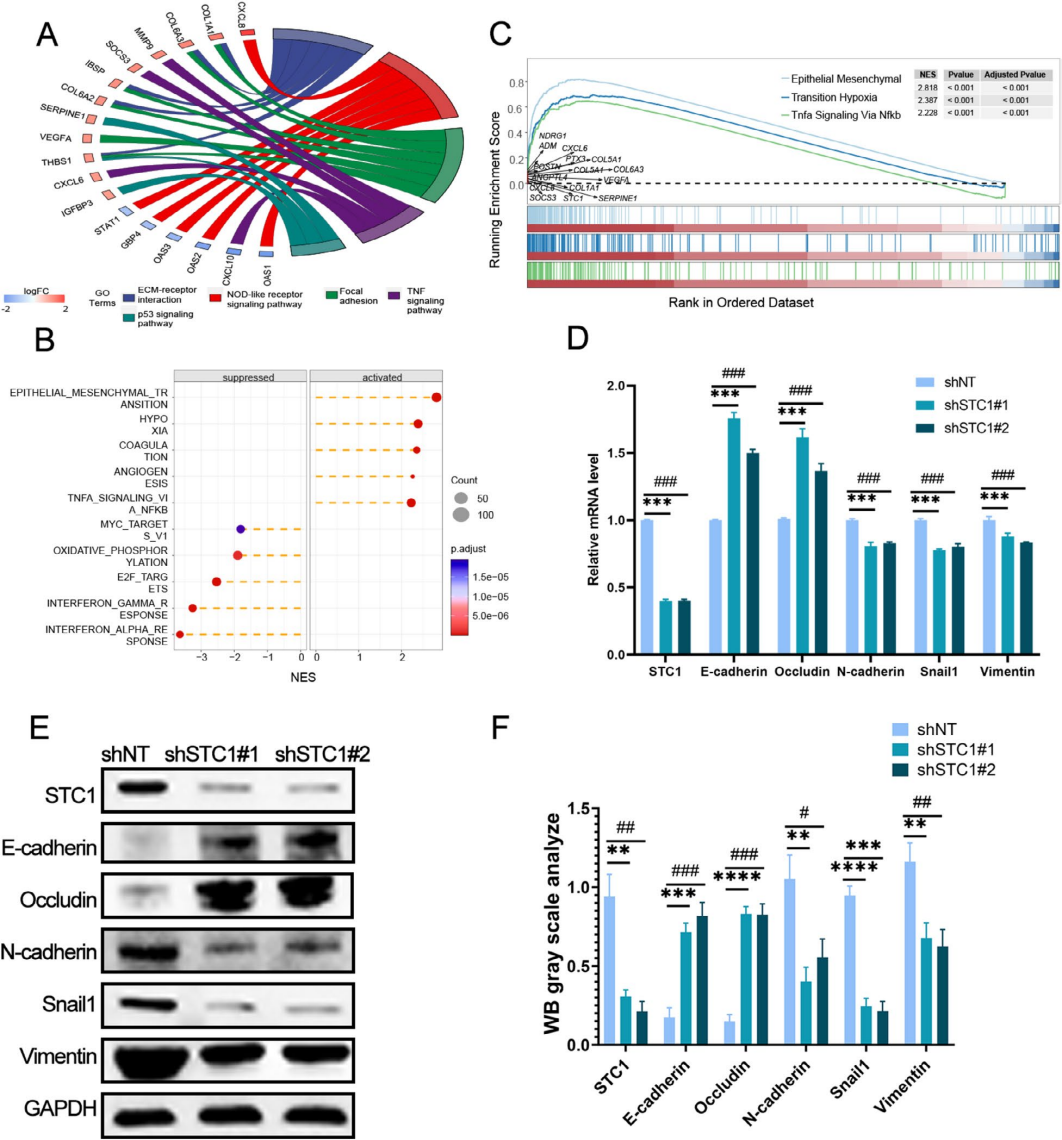
Knockdown of STC1 suppressed EMT in GBM cells. Gene enrichment analysis of expression‐correlated genes of STC1 by (A) GO, (B) KEGG, and (C) GSEA enrichment analysis. qRT‐PCR (D) and western blot (E) were employed to assess the expression of the correlated biomarkers of EMT in GBM cells knockdown of STC1 (***p < 0.001, ###*p* < 0.01, with Student's *t* test, *n* = 3). (F) Quantification of EMT marker protein expression by Western blot in STC1‐knockdown GBM cells (*/#*p* < 0.05, **/##*p* < 0.01, ***/###*p* < 0.001, ****/####*p* < 0.0001, with Student's *t* test, *n* = 3).

To further explore the potential regulatory mechanisms of STC1 on GBM, we performed bioinformatics analysis. The expression profiles of the GBM samples were extracted from the CGGA and TCGA databases and then divided into two groups depending on EXT1 expression. One group was STC1^High^ samples, and the other group was STC1^low^ samples. Hierarchical biclustering analysis was employed to screen the DEGs in the two groups. Subsequently, we conducted enrichment analysis of these DEGs. As shown in Figure 5A,B, the results of GO and KEGG enrichment showed that STC1 was involved in the EMT process and the NF‐κB pathway. The GSEA analysis was performed to verify the above results. The results also revealed that the EMT and NF‐κB pathways were enriched in STC1^High^ samples (Figure 5C). In addition, qRT‐PCR and western blot were used to measure the effect of STC1 on the expression of EMT‐associated proteins. The inhibition of STC1 increased the expression of epithelial markers (E‐cadherin and occludin). Conversely, the expression of mesenchymal markers (N‐cadherin, Snail1, and Vimentin) was downregulated in GBM cells transfected with shSTC1#1 or shSTC1#2 (Figure 5D–F, *p* < 0.001). In summary, these findings suggested that the knockdown of STC1 inhibited the EMT process in GBM cells.

### 3.6 Knockdown of STC1 Suppressed the Malignant Biological Behaviors of GBM Cells Through the NF‐κB Pathway

**FIGURE 6:**
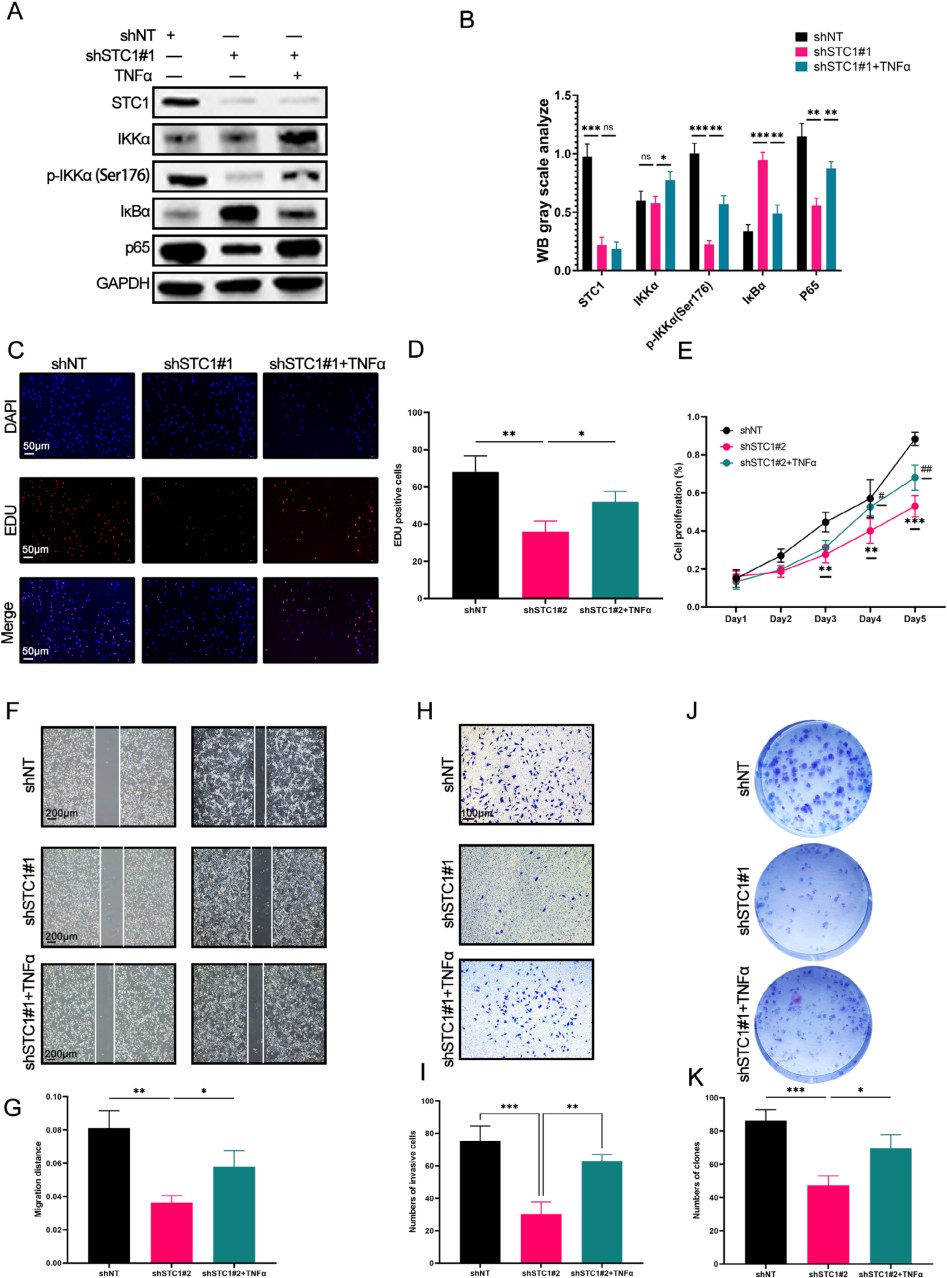
Silencing of STC1 inhibited the proliferation, migration, and invasion of GBM cells by NF‐κB signaling. The GBM cells were transfected with STC1 knockdown lentivirus and then treated with or without TNF‐α. (A) Western blot was performed to detect the expression of NF‐κB pathway‐related proteins. (B) Quantification of NF‐κB pathway‐related proteins expression by Western blot (ns = not significant, */#*p* < 0.05, **/##*p* < 0.01, ***/###*p* < 0.001, with Student's *t* test, *n* = 3). (C, D) EdU assay was used to detect the proliferation of GBM cells (**p < 0.01, ***p < 0.001, with Student's *t* test, *n* = 3). (E) CCK‐8 assay was used to detect the proliferation of GBM cells (**p < 0.01, ***p < 0.001, shNT vs. shSTC1#2, with Student's *t* test, *n* = 3; #*p* < 0.05, ##*p* < 0.01, shNT vs. shSTC1#2 + TNF‐α, with Student's *t* test, *n* = 3). (F, G) Wound healing assay was used to measure the migration of GBM cells (*p < 0.05, **p < 0.01, with Student's *t* test, *n* = 3). (H, I) Transwell invasion assay was utilized to detect the invasion of GBM cells (**p < 0.01, ***p < 0.001, with Student's *t* test, *n* = 3). (J, K) Colony formation test was performed to assess the proliferation of GBM cells (*p < 0.05, ***p < 0.001, with Student's *t* test, *n* = 3).

The above findings of enrichment analyses indicated that STC1 was also closely associated with the NF‐κB pathway. The results of western blot demonstrated that knockdown of STC1 increased the expression of IκBα and decreased the phosphorylation level of IKKα and the expression of p65 (Figure 6A,B), suggesting that the deletion of STC1 inhibited the NF‐κB pathway. Besides, EdU, CCK‐8, wound healing, transwell invasion, and colony formation assays were conducted to evaluate the proliferation, migration, and invasion in GBM cells. The results indicated that knockdown of STC1 suppressed the proliferation (Figure 6C–E,J,K, all *p* < 0.05), migration (Figure 6F,G, all *p* < 0.05), and invasion (Figure 6H,I, all *p* < 0.01) in GBM cells. Notably, the treatment of TNF‐α (an activator of the NF‐κB pathway) partially reversed the effects of sh‐STC1#1 (Figure 6A–J, all *p* < 0.05), implying that STC1 regulated the malignant phenotype of GBM cells by affecting the NF‐κB pathway.

## 4 Discussion

GBM (grade IV), one of the most common and aggressive malignant brain tumors, continues to have high incidence and mortality rates [27]. GBM patients often experience significant headache, dizziness, nausea, vomiting, neurological deficits, and other clinical profiles of intracranial hypertension [28]. Currently, TMZ, a novel oral alkylating agent, holds a pivotal position as the first‐line drug for glioma chemotherapy, particularly in GBM. However, its therapeutic efficacy is hindered by the development of drug resistance. MGMT is a key enzyme that repairs TMZ‐induced DNA damage by removing the methyl group from the O6 position of guanine, thereby reducing TMZ's cytotoxicity. The high expression of MGMT is a significant contributor to TMZ resistance [29]. MGMT inhibitors like O6‐benzylguanine (O6‐BG) have been explored to downregulate MGMT expression and enhance TMZ sensitivity [30]. However, their clinical use is limited due to high toxicity to normal cells [31]. Thus, it is essential to elucidate the resistance mechanism of TMZ for the treatment of GBM.

In the present study, we screened genes associated with TMZ resistance by bioinformatics techniques, including the construction of a cox regression model and NMF analysis. As well as STC1 was for study. Then, we validated STC1 expression in different databases. The results showed that STC1 was upregulated in GBM tissues and significantly associated with poor prognosis in GBM patients. Besides, the expression pattern of STC1 in GBM cell lines was consistent with the database. STC1 protein is initially discovered in fish, primarily produced by stannous bodies, and it is involved in regulating calcium/phosphorus balance [15]. STC1 is located on human chromosome 8p21.2, which frequently exhibits alterations in a number of cancers, suggesting that STC1 is associated with the development of tumors [32]. The expression and function of STC1 are often disturbed in pathological states, especially in cancer, where the expression level of STC1 is closely related to tumor progression and prognosis. It was reported that STC1 expression was upregulated in breast cancer, colorectal cancer, bladder cancer, and ovarian cancer, and this high expression was closely associated with the poor prognosis of cancer patients [33, 34, 35, 36]. Importantly, research pointed out that the expression of STC1 was also increased in GBM, and the proliferation, migration, and invasion of GBM cells were reduced when STC1 was absent [37]. Consistent with previous results, we similarly demonstrated that STC1 was overexpressed in GBM tissues and cell lines. The results of loss‐of‐function revealed that the knockdown of STC1 inhibited the proliferation, migration, and invasion of GBM cells, while STC1 overexpression promoted the malignant biological behavior and increased the TMZ resistance.

As we all know, EMT is considered to be the essential process in tumor cell metastasis and drug resistance [38]. EMT is the process by which epithelial cells lose their characteristic polarity and intercellular tight junctions in response to certain factors and transform into infiltrating mesenchymal cells [39]. Loss of epithelial cell phenotype and gain of mesenchymal properties are the main features of EMT occurrence, as evidenced by the expression of E‐cadherin decreasing and the expression of Vimentin and N‐cadherin increasing [40]. A study suggested that the expression of EMT markers in TMZ‐resistant cells was overexpressed, indicating that EMT was closely related to TMZ sensitivity in GBM cells [41]. In our work, the findings of enrichment analysis supported that DEGs related to STC1 were involved in the EMT pathway. In addition, western blot analysis showed that deletion of STC1 effectively inhibited the EMT in GBM cells. In previous studies, overexpression of STC1 stimulated the development of EMT in ovarian cancer cells, thereby promoting cancer cell metastasis [36]. The same regulatory mechanism was also found in human lung cancer; STC1 promoted the EMT process in cancer cells [42].

The results of GO, KEGG, and GSEA analysis also suggested that STC1 was enriched in the NF‐κB pathway. NF‐κB is present in almost all animal cells and responds to a wide range of stressors, including cytokines, radiation, heavy metals, and viruses [43]. Incorrect activation of NF‐κB has been connected with cancer, inflammatory and autoimmune diseases, infectious shock, viral infections, and improper immune development [44]. The activation of NF‐κB depends on the phosphorylation of IKKα, and following that activated IKKα induces other NF‐κB family members to form dimers and translocate into the nucleus, activating the expression of target genes [45]. The role of STC1 in the activation of the NF‐κB pathway has been widely demonstrated in a variety of cancers. Research has shown that overexpression of STC1 promoted NF‐κB phospho‐P65 (Ser536), thereby inducing apoptosis in cervical cancer cells, revealing STC1 as a potential therapeutic target for cervical cancer [18]. In addition, the similar function of STC1 was also observed in colorectal tumorigenesis [46]. However, the role of STC1 in the regulation of the NF‐κB pathway remained unclear in GBM. We first found that knockdown of STC1 inhibited the NF‐κB pathway in GBM cells, which was consistent with studies of STC1 in other cancers. Moreover, the inhibition of STC1 affected the proliferation, migration, and invasion in GBM cells through the NF‐κB pathway, which was partially reversed by the treatment of TNF‐α, further validating this view.

## 5 Conclusion

In conclusion, the current study confirmed that STC1 promoted the malignant biological behavior of GBM cells through the EMT and NF‐κB pathway, implying STC1 was an attractive target for the development of new therapeutic strategies for GBM.

## Author Contributions

Wanfu Xie contributed to the study conception and design. Jia Wang contributed to the writing of the first draft of the manuscript. Wei Wu contributed to the data collection and data analysis. Beichen Zhang and Haoyu Zhou contributed to doing the biological experiments. Bin Liu and Xiaobin Bai contributed to the formal analysis. Ruichun Li contributed to the software. All authors commented on previous versions of the manuscript, read, and approved the final manuscript.

## Ethics Statement

All utilization of data and samples in this study was approved by the Scientific Ethics Committee at the First Affiliated Hospital of Xi'an Jiaotong University (approve no. 2021‐695).

## Conflicts of Interest

The authors declare no conflicts of interest.

## Data Availability

The data used to support the findings of this study are available from the corresponding author upon request.
